# Non-Attended Representations are Perceptual Rather than Unconscious in Nature

**DOI:** 10.1371/journal.pone.0050042

**Published:** 2012-11-27

**Authors:** Annelinde R. E. Vandenbroucke, Ilja G. Sligte, Johannes J. Fahrenfort, Klaudia B. Ambroziak, Victor A. F. Lamme

**Affiliations:** Cognitive Neuroscience Group, Department of Psychology, University of Amsterdam, Amsterdam, The Netherlands; Radboud University Nijmegen, The Netherlands

## Abstract

Introspectively we experience a phenomenally rich world. In stark contrast, many studies show that we can only report on the few items that we happen to attend to. So what happens to the unattended objects? Are these consciously processed as our first person perspective would have us believe, or are they – in fact – entirely unconscious? Here, we attempt to resolve this question by investigating the perceptual characteristics of visual sensory memory. Sensory memory is a fleeting, high-capacity form of memory that precedes attentional selection and working memory. We found that memory capacity benefits from figural information induced by the Kanizsa illusion. Importantly, this benefit was larger for sensory memory than for working memory and depended critically on the illusion, not on the stimulus configuration. This shows that pre-attentive sensory memory contains representations that have a genuinely perceptual nature, suggesting that non-attended representations are phenomenally experienced rather than unconscious.

## Introduction

When watching a beautiful scene, for example when standing on top of a just conquered mountain, it might feel as if you are conscious of every element present in front of your eyes. This subjective impression, however, may be misleading. Studies using change detection have shown that when a scene is presented twice in succession, large changes often go unnoticed [1]. This has led researchers to suggest that only those parts of a scene that are attended are consciously perceived [2], [3], [4]. What happens then to the objects that are unattended? An obvious conclusion would be that momentarily unattended objects are unconsciously processed. However, recently it has been proposed that attention and consciousness are separate and maybe even orthogonal processes [5], [6], [7], [8]. Possibly, unattended objects are consciously processed, but not stored in working memory due to the bottleneck of attention. In this paper, we investigated the nature of unattended objects by assessing whether these objects carry hallmarks of conscious perception such as perceptual organization and perceptual inference, which are known to be absent during unconscious processing [9], [10].

The number of changes in a display that can be reported corresponds to the capacity of visual working memory, which has an average of 4 objects [11]. In the last few years, however, change detection research has shown that more changes can be detected when using a partial-report paradigm [12], [13], [14], [15], [16], [17]. If a specific location is cued *after* offset of a memory display, but *before* onset of the test display (with a so-called retro-cue), performance can be improved by as much as 300% [15]. This increase in performance is attributed to sensory memory [18], a form of memory that is established prior to attentional selection [17]. Sensory memory has a much higher capacity than visual working memory, but its representations are fragile and easily overwritten by new stimulation [14], [15]. Nevertheless, these representations can be retrieved for up to 12 seconds after stimulus offset [19], [20] and do not depend on eye movements that might guide attention before the cue [16], [21].

**Figure 1:**
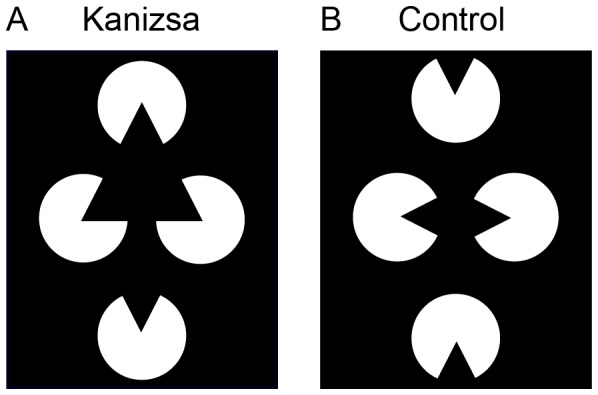
Two types of stimuli used in Experiment 1. In the Kanizsa condition (a) the inducers formed an illusory triangle. In the control condition (b) the same inducers were configured in such a way that no illusion was formed.

A consensus exists among researchers that representations in working memory have been consciously processed. It is still highly debated, however, whether sensory memory also consists of conscious representations [2], [22], [23], [24], [25], [26]. In this study we aimed to examine the nature of sensory memory by looking at its perceptual characteristics. In many cases, our perception of a stimulus deviates from the actual physical stimulus properties present in the scene. This process is called perceptual inference and is often studied using visual illusions. In the illusory Kanizsa figure [27], for example, an occluding surface can be inferred from the elements (inducers) in the scene (Fig. 1a). This surface is perceived as brighter than the background and has illusory contours that define its borders. The illusion thereby transforms the percept from meaningless fragmented input into a triangle lying on top of three disks. In this transformation, a percept is formed that actually moves away from the physical stimulus properties (i.e. disks are inferred that are not there). Recently, it was shown that the Kanizsa illusion requires conscious processing of the inducers [28]. When the inducers were made invisible by use of continuous flash suppression, subjects did not perceive the Kanizsa figure. In comparison, a simultaneous brightness contrast - the illusion of a white disc seeming brighter when presented on a black background than on a grey background - persisted even when the same procedure was used to make the surrounding context invisible. This implies that while simple contrast illusions are driven by low-level stimulation, perception of the Kanizsa figure depends on high-level inferential processes and represents a form of higher-order perceptual organization mediated by feedback interactions from higher to lower visual areas [29], [30], [31]. If figural information as present in the Kanizsa illusion could be maintained in sensory memory, this would imply that higher-order perceptual organization has occurred, which is in contrast with a description of sensory memory as being purely unconscious.

To study the presence of illusory figures in sensory memory, we embedded Kanizsa illusory triangles as objects in a cued change detection task. Two types of sensory memory - early and late - and working memory were measured using different cue timings (see Methods). Performance on the change detection task containing Kanizsa figures was compared to the same task containing control figures (Fig. 1b). We expected a beneficial effect for the Kanizsa figures compared to the control figures. If the Kanizsa illusion only influences the capacity of working memory, this would confirm that only working memory contains perceptually inferred representations. If, however, sensory memory benefits from the Kanizsa illusion as well, this would suggest that sensory memory is qualitative in nature and shares properties with working memory that are typical of conscious processing. Importantly, we predicted an interaction between figure condition and memory type. The capacity of sensory memory is known to be larger than the capacity of working memory [15], [18]. This larger capacity is established by the retro-cue benefit: performance on a change detection task exceeds working memory capacity when a retro-cue is presented. Therefore, if Kanizsa figures boost sensory memory capacity, it is expected that the boost for sensory memory will be larger than the boost found for working memory. This interaction would indicate that over and above a boost for working memory, there is an additional retro-cue benefit for illusory Kanizsa figures, demonstrating that sensory memory representations contain perceptual information.

## General Methods

### Subjects

Forty-three students of the University of Amsterdam (35 females and 9 males, age range 19–52) with normal or corrected-to-normal vision participated in this study. Thirty-nine participants (30 females and 9 males) passed the training criterion (see Procedure) and either participated in the first experiment (20 subjects, age range 19–51, M = 25, SD = 7) or in the second experiment (20 subjects, age range = 19–51, M = 24, SD = 8; 1 participant participated in both experiments). The local ethics committee of the department of Psychology of the University of Amsterdam approved the experiment and all subjects gave their written informed consent.

### Task

**Figure 2:**
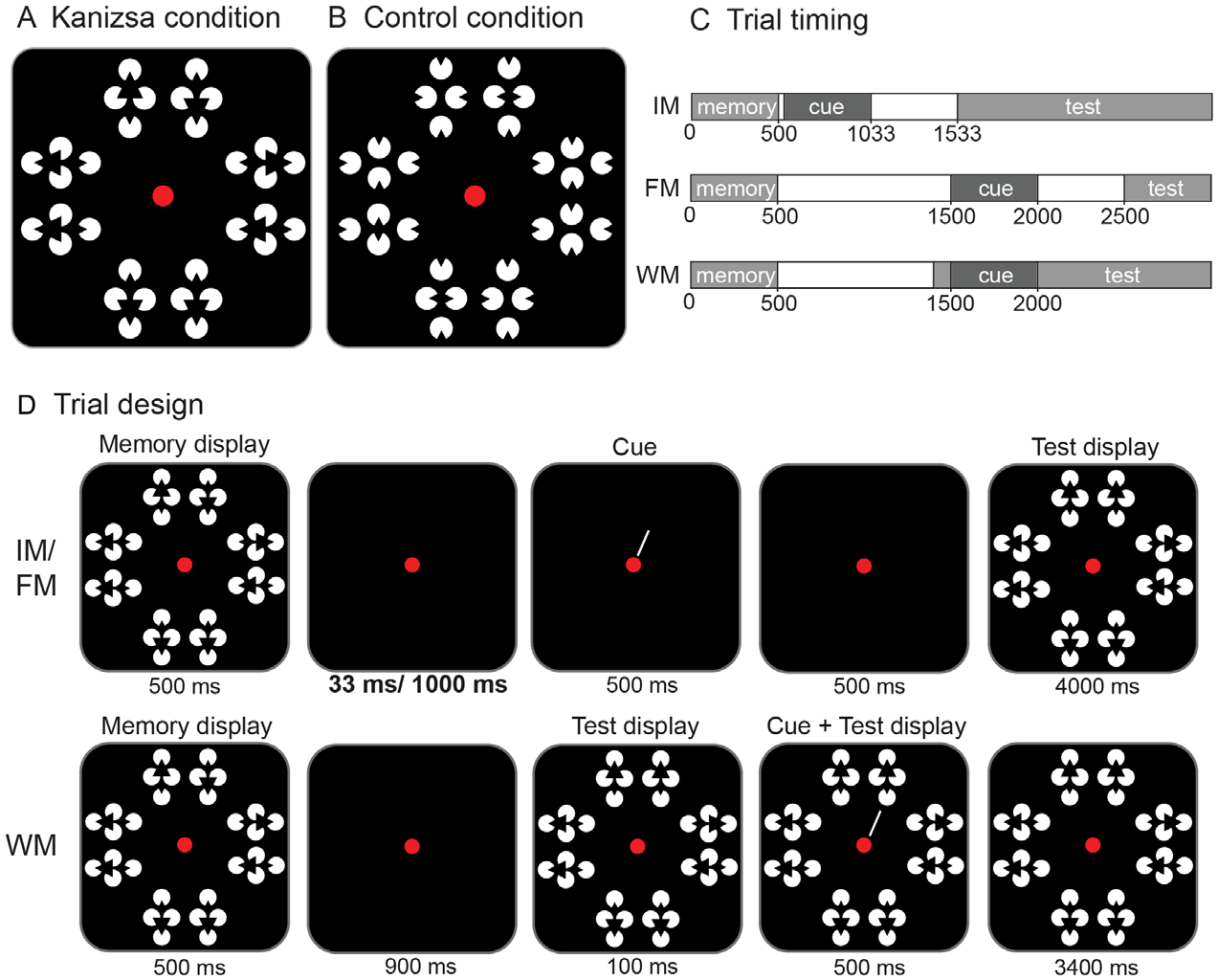
Configuration of the stimulus display for the Kanizsa condition (a) and the control condition (b). The 8 stimuli were placed radially around the fixation dot in both conditions. A change (50% of the trials) always involved a rotation of the two middle inducers of one stimulus. The trial timings are depicted in (c) and the stimulus displays in (d). There were two sensory memory conditions: Iconic Memory (IM) and Fragile Memory (FM). In these two conditions, the cue was presented after the memory array, but before the test array. In the Working Memory condition (WM), the cue was presented just after presentation of the test array. Sensory memory is erased by the test array.

Subjects were asked to fixate on the red dot in the center of the screen throughout each trial. The red dot turned green for 1000 ms to indicate the start of a trial. Then, a memory display containing eight figures appeared for 500 ms (Fig. 2). Subjects were instructed to remember as many objects in this memory display as possible. On each trial, one figure was cued by a line (500 ms) that singled out the figure that could change. All non-cued figures remained the same between memory and test display and the cue was always valid. During presentation of the test display, subjects indicated by button press whether the cued figure was the same (50% of the trials) or different (50% of the trials) compared to the memory display and it was stressed to respond “no-change” when subjects were uncertain about their choice. After the response, subjects received auditory feedback on their performance on that trial.

To separate the capacity for sensory memory and working memory, three types of trials were presented (Fig. 2c and d). Two different cue timings were used to measure sensory memory. On early sensory memory trials, from now on termed the iconic memory condition (IM [32]), the cue was presented 33 ms after offset of the memory display. A 500 ms blank screen separated the cue and the test display to allow the cue to be fully processed and to prevent interference with the test display. Previous studies have shown that cues presented immediately after offset of the memory array tap into a form of sensory memory that is dependent on afterimages [15]. When, for example, a light mask is presented before the cue this reduces the high performance to a performance level found for late (>1000 ms) cue timings. On late sensory memory trials, from now on termed the fragile short-term memory condition (FM [15]), the cue was presented 1000 ms after offset of the memory display. Therefore, the representations had to be maintained in memory for a 1000 ms interval before attentional selection came into play (note that with such an interval between stimulus offset and cue, any account of the cue directly affecting the representation of the memory display becomes invalid [26]). As retinal persistence and phosphor persistence of the monitor do not last for 1000 ms, performance in this condition cannot purely depend on retinal afterimages anymore but has to depend on a more genuine form of memory (or ‘neural afterimage’). Again, the test display was presented 500 ms after cue offset. On working memory trials (WM [11]), the test display was presented 900 ms after offset of the memory display. The cue then appeared 100 ms after onset of the test display and remained on screen together with the test display for 500 ms. This way, the memory display and any retinal or ‘neural’ afterimage of it (IM and FM) was overwritten by the test display and only those items of the memory display that had been attended and robustly stored in WM remained available for detecting a potential change. In both the FM and WM trials, the lag between memory display offset and cue onset was 1000 ms. In all conditions, the test display remained on screen for 4000 ms or until the subject made a response.

### Procedure

In the first session, subjects were trained on the basic version of the change detection task containing white rectangles instead of the inducer elements. The training consisted of blocks of 60 trials in which IM, FM and WM conditions were intermixed. Subjects practiced for a minimum of 4 and a maximum of 12 blocks of 60 trials. Only subjects that reached a performance level of 75% were allowed to enter the next phase of the experiment. This criterion was incorporated to prevent participants performing at chance level in the experimental session, which was predicted to be more difficult. After the basic change detection task training, subjects were trained on the actual experiment containing the Kanizsa figures for 4 blocks of 72 trials (24 trials per condition, randomly intermixed within blocks: memory-type (3) × figure-type (2)) resulting in a total of 288 trials. No performance level criterion was used for this task.

In the second experimental session, which took place after a minimum of 30 minutes rest or a maximum of 14 days later, subjects first performed 12 practice trials of the Kanizsa change detection task (two trials per condition: memory-type (3) × figure-type (2)). Then, subjects performed 12 blocks of 72 trials (24 trials per condition, randomly intermixed within blocks: memory-type (3) × figure (2)), making a total of 864 trials. Performance on these 864 trials was analyzed using a 3 (memory-type) × 2 (figure) Repeated Measures ANOVA. Post-hoc analyses showed that there was no correlation for the interval between training and experimental session and the average performance on the experimental session (Exp 1: Pearson’s R = −.23, p = .32; Exp 2: Pearson’s R = .16, p = .51).

## Experiment 1

In the first experiment, the performance on a change detection task containing Kanizsa figures was compared to a control condition in which Kanizsa inducers were presented that did not induce an illusory percept. In both conditions, the change in any object could be perceived by virtue of the fact that two inducers of that object changed (see Fig. 2a and b). However, in the Kanizsa condition, an illusory percept was induced that could aid the subject by remembering triangles that point towards the center of the screen or away from it.

### Methods

#### Stimuli

During the training session, a basic version of the change detection task was performed [15], in which the figures consisted of white rectangles oriented horizontally, vertically, 45° to the vertical, and 135° to the vertical. Subjects were shown memory and test displays containing eight rectangles (1.6°x 0.45°) placed radially within 2.29° from a red fixation dot on a black background. The cue was a 3-pixel thick line (2° x.05°) pointing towards one of the eight locations.

In the second part of the training and in the actual experiment, two sets of stimuli were used (outline of stimuli: 2° × 3.15°), which constituted an experimental (Fig. 2a) and a control condition (Fig. 2b). Both sets consisted of four white inducers (circle radius of.42°, gap width of.45°). As in the basic version of this task, subjects were shown memory and test displays containing eight figures from one of two possible sets, placed radially around the red fixation dot at 2.29° distance. In the experimental set (Kanizsa condition) the inducers formed a Kanizsa triangle pointing towards or away from the fixation (size triangle: 1.15° × 1.13°). The support ratio (the ratio of the physically specified contours to the total contour length) of the Kanizsa triangle was 0.67. In the control condition inducers formed figures with the middle inducer rotated inwards or outwards compared to the center of the figure itself. All stimuli were white, presented on a black background.

### Results

**Figure 3:**
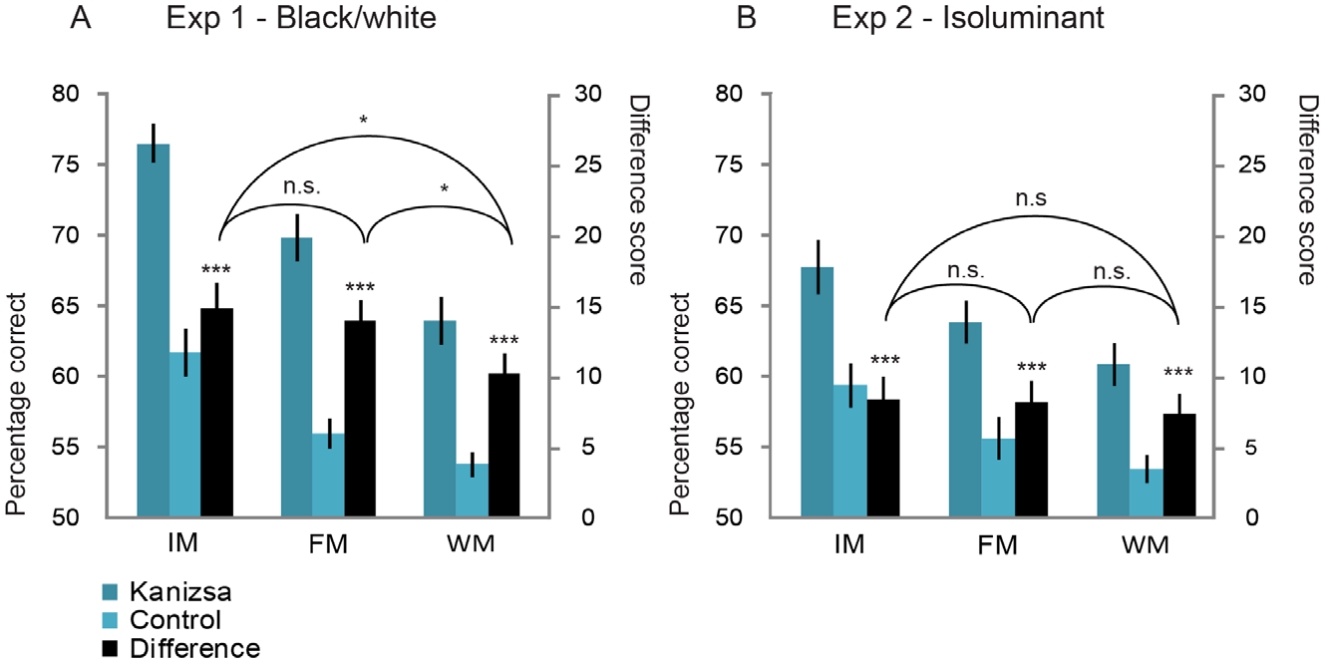
Mean percentage correct for Experiment 1 (a) and 2 (b). In Experiment 1 (a), performance on all memory types was higher for the Kanizsa condition (dark blue) compared to the control condition (light blue; difference scores in black). Importantly, the boost for sensory memory (IM and FM) was larger than the boost for working memory (WM). In Experiment 2 (b), performance for the Kanizsa condition was again higher compared to the control condition, but the boost was the same for each memory type. Error bars depict standard errors. ***p<.001; *p<.05.

Figure 3A shows the average performance in percentage correct for the Kanizsa and control condition for each type of memory. IM performance was higher than FM performance, which was higher than WM performance, F(2,38) = 64.5, p<.001, η^2^ = .773. This confirms that sensory memory capacity (IM and FM) is larger than WM capacity, consistent with previous findings [15], [16], [17], and that an early cue as used in the IM condition enhances performance more than a late cue as used in the FM condition [15]. Also, performance in the Kanizsa condition was higher than performance in the control condition (F(1,19) = 123.6, p<.001, η^2^ = .867). This suggests that the added percept of a triangle present in the Kanizsa condition aided subjects’ memory.

Importantly, the benefit for the Kanizsa versus the control condition differed for the three types of memory (F(2,38) = 3.8, p = .032, η^2^ = .165). Post hoc t-tests showed that the difference between the Kanizsa and control condition was larger for IM and FM (14.8% and 13.9%, respectively) than for WM (10.1%; Fig. 3A; t(19) = 2.3, p = .034, t(19) = 2.4, p = .029). This indicates that IM and FM had a larger benefit from the illusory percept than WM did, as was expected based on the larger capacities for sensory memory than for working memory in general.

As our subject pool had a fairly wide age range, we added age as a covariate to the Repeated Measures ANOVA to see whether the interaction effect could be explained by age. Also, there was quite some variability in the interval between the training and the actual experiment (see Methods), which could have influenced the results. However, neither age (F(2,34) = 1.7, p = .204) nor train-test interval interacted with the significant memory type × figure effect (F(2,34) = .2, p = .814) or any of the main effects (all F <1.9, all p>.171).

Control analyses confirmed that the interaction effect could not be accounted for by a possible floor effect in the WM condition (which would be chance performance of 50%). When splitting up the group in high- and low-WM control performers (M = 54.8% versus 52.7%), the interaction effect remained in both groups. Although statistical testing for an interaction effect on 10 subjects yields too little power, we found the same numeric increase in performance on Kanizsa trials for both the high and the low performers (low: IM –15.1%, FM –13.8%, WM –10.1%; high: IM –14.5%, FM –14.0%, WM –10.2%). Together with the fact that the WM control condition differed significantly from chance (post-hoc t-test: t(19) = 4.9, p<.001), these findings suggest that a floor effect could not explain our results.

### Discussion

In Experiment 1, we investigated the performance for Kanizsa figures in IM, FM and WM. The results clearly show that all types of memory benefit from the Kanizsa illusion compared to the control condition. Moreover, IM and FM exhibited a larger benefit from the perceptual nature of the Kanizsa condition than WM did. This shows that apart from the working memory component that contributed to the performance in the sensory memory conditions, there was a retro-cue benefit in the Kanizsa condition that exceeded the benefit resulting from the retro-cue in the control condition This suggests that the Kanizsa illusion was already represented in sensory memory, enabling an extra boost to the performance on these conditions over and above the boost that is seen without the Kanizsa illusion. As the Kanizsa illusion is a prime example of perceptual inference, which depends on conscious perception [9], [28], [31], these findings are a first indication that IM and FM consist of qualitative representations. To investigate whether the boost in performance seen in the Kanizsa condition truly depends on the perceptually inferred characteristics of the Kanizsa illusion, a second experiment was conducted in which the configuration of the elements remained the same, but the strength of the illusory percept was largely reduced.

## Experiment 2

**Figure 4:**
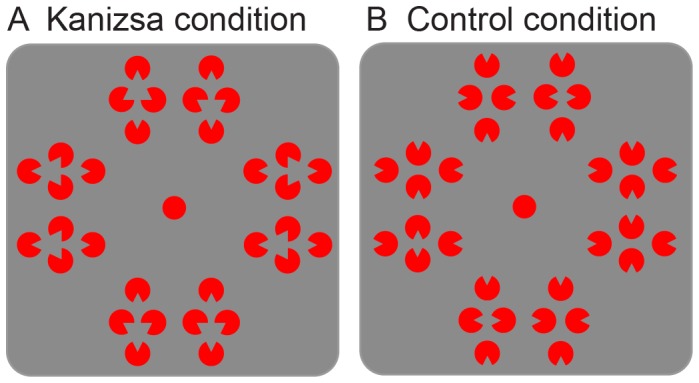
Stimulus displays for Experiment 2. The background was made subjectively isoluminant with respect to the bright red inducers for each participant. This reduced the illusion in the Kanizsa condition (a) while the control condition (b) stayed perceptually the same as in Experiment 1.

In Experiment 2, the effect of perceptual inference in the Kanizsa illusion on memory performance was investigated. Instead of using black-and-white Kanizsa figures, the inducers were made isoluminant with respect to their background. This has been shown to decrease the Kanizsa illusory effect [33], [34], an effect than can to some extent be observed from Figure 4 (when properly fixating the fixation dot and when the screen on which it is viewed shows the red and grey as isoluminant). Although perceptual organization of the elements still enables the formation of a triangle, the perceptual quality of illusory contours and the illusory contrast difference between the region of the triangle and its surround disappear under conditions of isoluminance. The crucial difference with Experiment 1 is therefore that the triangle can only be cognitively inferred and the perceptually inferred characteristics are heavily diminished. The difference between cognitive inference and perceptual inference of the triangle is whether you decide there should be a triangle compared to actually perceiving a triangle, similar to for example knowing that a car has moved because the second time you see it, it is in a different location versus actually perceiving the movement of the car [35]. If the boost in performance for the Kanizsa condition found in Experiment 1 was due to the added features and thus the perceptual aspects of the figure, than the interaction effect between figure condition and memory type would disappear for the isoluminant Kanizsa figures in Experiment 2.

### Method

#### Stimuli

The experiment was the same as Experiment 1, except now the stimuli were red inducers on an isoluminant, grey background (24 cd/m^2^, see Fig. 4). At the start of the experimental session, the background was set to subjective isoluminance for each participant by using a Flicker Photometry task [36]. In this task, a checkerboard pattern consisting of red and grey squares alternating at a frequency of 30 Hz was presented. The RGB value of the red squares was kept constant (brightest possible: 255,0,0) and subjects were instructed to adjust the color of the grey squares in RGB color space by pressing one of two buttons until a minimum amount of flicker was observed. The Flicker Photometry task was presented three times and the average RGB value was taken as color value for the background of the display.

### Results

Figure 3B shows the performance in percentage correct on the isoluminant Kanizsa and control conditions for each memory type. As in Experiment 1, a main effect of memory type was found (F(2,38) = 28.0, p<.001, η^2^ = .596). This indicates that performance in the sensory memory conditions was again better than performance in the WM condition. Also, performance in the Kanizsa condition was higher than performance in the control condition, (F(1,19) = 44.0, p<.001, η^2^ = .698). Apparently, even when the illusory nature of the effect is largely removed, merely organizing the inducers in such a way that enables one to cognitively infer the presence of triangles is sufficient to boost memory capacity. Crucially, however, the interaction between memory type and figure condition was absent (F(1.5,31.6) = .3, p = .677, η^2^ = .016, Greenhouse-Geisser corrected) indicating that there was a benefit for the isoluminant Kanizsa condition versus the control condition, but this boost in performance was the same for all three memory types (IM: 8.5%, FM: 8.2%, WM: 7.5%). As in Experiment 1, we added age and days between training and experimental session as covariates in our analyses and found no significant effects (all F <3.2, all p>.057).

To compare the results of Experiment 2 to Experiment 1, experiment was implemented as a between-subject factor in the Repeated Measures ANOVA. The three-way interaction between memory type, figure condition and experiment was not significant (F(2,76) = 1.4, p = .256, η^2^ = .035), indicating that the direction of the interaction effect between memory type and figure condition was the same in both experiments. However, the lack of the three-way interaction could have been due to low statistical power (.289). As our hypotheses predicted an interaction between VSM and VWM, we combined the IM and FM condition and tested the two VSM conditions averaged against VWM. This resulted in a marginally significant interaction between memory type, figure condition and experiment (F(1,38) = 3.2, p = .081, η^2^ = .078). When the same interaction effect was tested using a Monte-Carlo permutation test (1,000,000 iterations of reshuffling the data), there was a just significant effect (p = .048). These two analyses suggest that the direction of the interaction effect was as expected: for Experiment 2, the difference between Kanizsa and control was smaller than for Experiment 1, especially for the VSM conditions.

In addition, when looking at the results for the control condition and Kanizsa condition separately, it can be seen that memory performance on the control condition was similar for the two experiments (Exp. 1: IM 61.7%; FM 56.0%; WM 53.8%; Exp 2: IM 59.4%; FM 55.7%; WM 53.5%), while memory performance on the Kanizsa condition differed (Exp 1: IM 76.5%; FM: 69.9%; WM: 63.9%; Exp. 2: IM 68.0%; FM 63.9%; WM: 60.9%). We therefore tested the experiment effect for the two figure conditions in separate Repeated Measures ANOVAs. The Repeated Measure Analysis for the control condition showed that there was no interaction between experiment and memory Type (F(1.7,65.2) = .87, p = .41, η^2^ = .022, Greenhouse-Geisser corrected), while for the Kanizsa condition there was a significant interaction (F(2,76) = 5.5; p = .006, η^2^ = .127). This suggests that when comparing the experiments, performance on the control condition was the same for all three memory types while performance on the Kanizsa condition differed between memory types.

### Discussion

In Experiment 2, the effect of the added percept in the Kanizsa illusion was removed by making the figures isoluminant. Although keeping a form of spatial organization - and thus cognitive inference - intact, this severely diminished the perceptual illusion of an occluding figure surface; logically, the only figure that the combination of inducers can make is a triangle (cognitive inference), but the actual visual aspects of the figure (perceptual inference), i.e. its surface and contours, are absent. Under isoluminance, a beneficial effect for the Kanizsa figures versus the control figures was found for all memory types. Crucially, however, the interaction effect between sensory memory and working memory disappeared. This suggests that the extra boost for sensory memory found in Experiment 1 relied on the perceptual illusory aspects present in the black-and-white Kanizsa figures and not on cognitive inference induced by the spatial configuration itself.

## General Discussion

In this study, we examined the underlying nature of sensory memory representations. It is highly debated whether sensory memory representations are fragmented and unconscious [2], [23], [25], [26] or whether they are qualitative in nature and therefore phenomenally conscious [22], [24]. To investigate the characteristics of sensory memory, the effect of a Kanizsa illusion on sensory memory performance was examined. In this Kanizsa illusion, a set of inducers was aligned in such a way that they formed an illusory occluding triangle defined by illusory contours and a contrast difference between surface and background. The Kanizsa illusion was shown to have a beneficial effect on sensory memory compared to a control condition. Importantly, in Experiment 1 the boost for sensory memory (IM and FM) was larger than the boost for WM, as was predicted based on their different baseline capacities. This shows that the boost in the sensory memory conditions was not only driven by a potentially shared working memory component between conditions; sensory memory had a larger increase in performance from the Kanizsa illusion than working memory had from the Kanizsa illusion, showing that there is a benefit in performance over and above the advantage of the illusion that is seen in working memory. In Experiment 2, however, the figures were made isoluminant - keeping the spatial organization that enabled cognitive inference intact while removing the percept of illusory contours and the illusory contrast difference between surface and background – and the interaction effect between sensory memory and WM disappeared. This suggests that the extra boost found for sensory memory depended on the perceptual quality of the original Kanizsa figures and not merely on spatial organization or attentional selection.

An important aspect of why the perceptual quality of the Kanizsa figure should be termed phenomenological is that to perceive the Kanizsa figure, higher-level inference is necessary, which does not occur when the inducing elements are unconsciously processed [28]. In the illusion, the percept moves away from the physical stimulus properties in the sense that objects are perceptually inferred that are not physically present in the image. This is in contrast to subliminal priming for example, in which semantic and categorical aspects of the stimulus can be extracted [37], but the meaning of the stimulus is processed as it is, and is not altered by inference mechanisms. Whether the semantic processing of words or pictures might be called qualitative as well is of course still open for debate. However, the mechanisms at play in the Kanizsa illusion that allow perception of the exact figure composition (e.g. whether the illusory figure is pointing towards the fixation point or away from it, see following paragraph) are likely to depend on conscious processing [28].

Although evidence has been found that the Kanizsa illusion is not perceived when its inducers are made invisible [28], other studies have found that the Kanizsa illusion survives crowding [38] and breaks through interocular suppression more easily [39], suggesting that processing of the Kanizsa illusion can occur unconsciously or preconsciously. On the one hand, these studies seem to contradict each other, but it might be that the formation of the Kanizsa illusion is dependent on a diverging set of mechanisms. The critical manipulation in the masking study [28] was that subjects had to indicate which direction the Kanizsa triangle was facing, while in the interocular suppression study [39], subjects merely had to detect the presence of the stimulus on the left or right side of the screen. In the latter study, basic grouping mechanisms that are known to depend on fast, feedforward activity [40] might have driven the easier break through. It could well be that explicit figure formation depends on later, recurrent activity and this latter process is associated with conscious processing [8], [28], [39]. However, since evidence about the requirement of conscious processing for perceiving the Kanizsa illusion is not clear-cut (such as in the case of crowding [38]), we cannot unequivocally claim that processing of the Kanizsa figures in sensory memory implies that sensory memory reflects conscious processing. More research on the processing of Kanizsa figures is needed to strengthen this claim.

The retro-cue that was used in this study is thought to guide attention to one of the representations in sensory memory and thereby make this representation robust to interference from the test display and available for report. It could therefore be argued that these representations are initially fragmented and unconscious, and attention is necessary to form a coherent and conscious percept [2], [26]. In this study, however, that does not seem to be the case. If attention was the sole factor for the Kanizsa illusion to become coherent and qualitative, the interaction effect found in Experiment 1 cannot be explained: if the cue determines the formation of the illusion, the boost in performance should be the same for each cue condition. Our results show that the Kanizsa illusion was already present in the representation of the array before arrival of the cue. Moreover, electrophysiological studies have shown that perceptual organization and figure-ground segmentation form the basis for selective attention and attention spread [41], [42], suggesting that attention depends on the structure provided by perceptual organization rather than the other way around. In Experiment 2, on the other hand, the interaction effect was not found. There seemed to be a basic advantage of the stimulus configuration when grouping Kanizsa elements together even when they did not result in a concurrent perceptual illusion. This advantage may have indeed depended on attention, and is therefore the same for the working memory and retro-cue conditions; in all conditions – also the IM and FM conditions - a working memory component is measured. During the memory array, there will always be some items that are attended and stored in WM. The iconic and fragile memory components thus ride on top of the WM capacity. In Experiment 2 it was shown that the added capacity for the Kanizsa configuration was not different for the three conditions. This implies that the results are explained by the benefit of cognitive inference that occurs in working memory and are mediated by attention. However, attention cannot explain the effect of the Kanizsa illusion for the retro-cue conditions in Experiment 1, in which there was an additional benefit on top of the benefit found for the working memory condition.

The presented results suggest that the representations underlying sensory memory are phenomenological. This seems in contrast with a recent study using a similar paradigm, in which it was suggested that the rich phenomenology experienced outside the focus of attention might be a false impression [43]. When asked to remember a display containing three rows of four letters, subjects failed to detect a pseudo-letter presented in one of the uncued rows. This may be interpreted such that the representations held in sensory memory are fragmentary and therefore unconscious. If sensory memory had been conscious at the moment of perception, the pseudo-letters should have been remembered. However, the design used by de Gardelle et al, was not optimized to measure sensory memory: they presented a mask after offset of the memory array, potentially abolishing sensory memory before the cue was presented. This interpretation is supported by the fact that they measured an average sensory memory capacity of 1.47 letters per row, which is much lower than the 3 items found in the traditional Sperling experiment [18]. Moreover, the recall procedure for the pseudo-letters tapped into the uncued row, while the crux of measuring sensory memory is that the specific row needs to be cued to be able to report about it. Therefore, the study by de Gardelle et al. mainly demonstrates that illusions can occur during the brief presentation of visual displays, rather than refuting the claim that sensory memory has a high capacity.

In the current study, capacity is used as a measure to draw conclusions about the perceptual nature of unattended representations. However, we do not want to claim that memory capacity in itself is directly related to phenomenology. Rather, we show that a perceptual property of the Kanizsa illusion is able to boost the capacity of sensory memory, and that hence sensory memory holds items with some sort of perceptual status. Obviously, to warrant the conclusion that sensory memory holds items in a fully perceptual or even conscious status would require more evidence. Many dimensions of perceptual quality would have to be compared between sensory memory and unequivocally conscious representations, either psychophysically or using neuroimaging techniques. Only when sensory and attended or accessed representations are similar in many or all perceptual dimensions the conclusion is warranted that sensory memory is a remnant of conscious vision. We have merely added a single dimension in that investigation, supporting the growing debate on the conscious or unconscious nature of sensory memory [2], [22], [23], [24], [25], [26]. Our results match with previous work, in which it was shown that sensory memory does not only support change detection, but can also be used for identification of real-life objects [44]. The results are also in line with a recently published study that showed the presence of the Ponzo and Ebbinghaus illusion in iconic memory [45]. This implies that sensory memory does not merely entail simple features - such as orientation - that can also be represented unconsciously [46], [47], but consists of higher-level integrated representations with a phenomenological basis. This potentially places pre-attentive sensory memory representations in the domain of phenomenal consciousness and outside the domain of unconscious processing.
